# Improving Real-Time Position Estimation Using Correlated Noise Models †

**DOI:** 10.3390/s20205913

**Published:** 2020-10-20

**Authors:** Andrew Martin, Matthew Parry, Andy W. R. Soundy, Bradley J. Panckhurst, Phillip Brown, Timothy C. A. Molteno, Daniel Schumayer

**Affiliations:** 1 Department of Physics, University of Otago, 730 Cumberland St, Dunedin 9016, New Zealand; andrew.martin@otago.ac.nz (A.M.); souan326@student.otago.ac.nz (A.W.R.S.); panbr907@student.otago.ac.nz (B.J.P.); phillb@elec.ac.nz (P.B.); tim@physics.otago.ac.nz (T.C.A.M.); 2 Department of Mathematics and Statistics, University of Otago, 730 Cumberland St, Dunedin 9016, New Zealand; mparry@maths.otago.ac.nz

**Keywords:** GPS, uncertainty quantification, sensor fusion, noise models, embedded computing, system performance evaluation, positioning algorithms

## Abstract

We provide algorithms for inferring GPS (Global Positioning System) location and for quantifying the uncertainty of this estimate in real time. The algorithms are tested on GPS data from locations in the Southern Hemisphere at four significantly different latitudes. In order to rank the algorithms, we use the so-called log-score rule. The best algorithm uses an Ornstein–Uhlenbeck (OU) noise model and is built on an enhanced Kalman Filter (KF). The noise model is capable of capturing the observed autocorrelated process noise in the altitude, latitude and longitude recordings. This model outperforms a KF that assumes a Gaussian noise model, which under-reports the position uncertainties. We also found that the dilution-of-precision parameters, automatically reported by the GPS receiver at no additional cost, do not help significantly in the uncertainty quantification of the GPS positioning. A non-learning method using the actual position measurements and employing a constant uncertainty does not even converge to the correct position. Inference with the enhanced noise model is suitable for embedded computing and capable of achieving real-time position inference, can quantify uncertainty and be extended to incorporate complementary sensor recordings, e.g., from an accelerometer or from a magnetometer, in order to improve accuracy. The algorithm corresponding to the augmented-state unscented KF method suggests a computational cost of $O(d_{x}^{2}d_{t})$, where $d_{x}$ is the dimension of the augmented state-vector and $d_{t}$ is an adjustable, design-dependent parameter corresponding to the length of “past values” one wishes to keep for re-evaluation of the model from time to time. The provided algorithm assumes $d_{t}=1$. Hence, the algorithm is likely to be suitable for sensor fusion applications.

## 1. Introduction

Global Positioning System receivers output a time-series of position measurements, but this signal suffers from errors due to many reasons, such as atmospheric and multipath effects [1,2,3] or insufficient coverage of satellite constellations. Rather than providing a full quantification of uncertainty, GPS devices generally provide Dilution-Of-Precision (DOP) variables, dimensionless multiplicative factors of the position error, designed to flag bad satellite coverage. In this paper, we develop algorithms that issue real-time position predictions with simultaneous uncertainty quantification and evaluate their performance. Embedding these algorithms in devices that use single frequency GPS will allow such devices to supply improved position estimates and real-time estimates of their uncertainty. Additionally, we will show in Section 5 that the DOP variables provide a negligible improvement to the error estimation.

Kalman filters [4] are commonly used to estimate the state of a system from a time-series of measurements corrupted with uncorrelated Gaussian noise. Such filters are promising candidates to infer position from GPS measurements and to characterise the uncertainty of the position estimates. However, previous studies have shown GPS position errors to be temporally correlated, due to environmental processes such as multipath and atmospheric effects [1,2]. These processes are too complicated either to model or to approximate in real time; thus, we opted to capture and then model the noise characteristics. A recent study found several correlated noise models [5,6,7], including the well-known Ornstein–Uhlenbeck (OU) model, to be a superior representation of GPS noise to the independent and identically distributed (iid) Gaussian noise model [8]. The correlated noise necessitates an extension to the ordinary KF.

Kalman filters have been extended by shaping filters to deal with correlated noise [1,2,9,10]. Such augmented filters have allowed improved measurement in the difference in position between two dual-frequency GPS receivers, one of which has an adjustable height [1,2]. The differential signal studied in References [1,2] still had correlated noise, but with a different character than that of a stand-alone single-frequency receiver, including a much reduced noise amplitude and shorter coherence time. The shaping filter included a noise variable governed by an Ornstein–Uhlenbeck (OU) process [11]. The authors found the inclusion of OU noise to be beneficial for detecting the changes in distance between the receivers; however, the study did not address the position uncertainty, which is the focus of the current paper.

Since the KF is a class of sequential Bayesian inference algorithms, the uncertainty in the position measurement at each time-step is given by the shape of the posterior distribution. The quality of probability forecasts is commonly compared using scoring rules [12,13,14,15,16], which allow the quantification of probability distributions based on both their accuracy (how closely centred near the “true” value) and precision (how tightly they are distributed). In order to compare the posteriors from different algorithms, we use the log score [15,16]. The log scores allow us to evaluate the effect of the noise model on uncertainty quantification of position inferences on GPS time-series datasets and quantify the improvement gained by using the OU noise model.

We developed stochastic algorithms with different noise models to predict the position of a stand-alone single-frequency GPS receiver and quantified the uncertainty of its position. We evaluated the performance of the KF algorithms, as well as a basic, non-learning method, which uses no filtering. Based on a scoring method, developed for comparing probability distributions, we ranked the algorithms for performance and assessed the importance of the dilution-of-precision from incomplete satellite coverage. The OU noise-model augmented with a KF seemed superior than all other models discussed. However, the OU noise model did not reach the same performance with altitude inference and scored worse. We note that we carried out all modelling on a desktop computer, hence the algorithm in Appendix A in its current state is only suitable for a similar implementation. The computational cost of this algorithm is minimal, of the order of $O(d_{x}^{2}d_{t})$ where $d_{t}$ is determined by a design choice and can be as low as unity, while $d_{x}$ is the dimension of the augmented state-vector; hence, it can be of the order of 10. One of the challenges one may face with in implementing the algorithm in hardware is the parameter’s potential dependence on geographic location. Investigating this potentially weak dependence is left for future research.

The structure of the paper is as follows: In Section 2, we analyse the GPS signals, fitting noise model parameters while observing their variation with respect to global position and coordinate type (latitude, longitude and altitude). The well-characterised noise allows us to construct well-informed models and prior distributions for the variables used in KF algorithms in Section 3. In Section 4, we formulate the scoring rules to evaluate algorithm performance. In Section 5, we present the results of the inferences and uncertainty quantification. We conclude in Section 6.

## 2. Data Analysis

**Figure 1:**
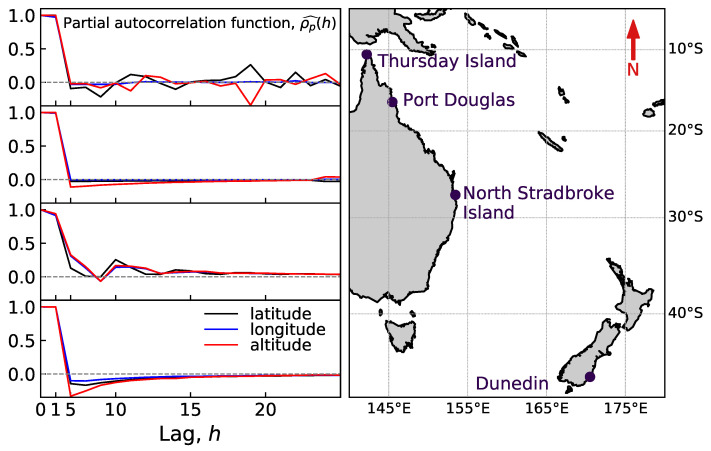
Map of GPS receiver locations for the datasets considered. A single GPS receiver was located at different times at the labelled locations: Dunedin (New Zealand) and North Stradbroke Island, Port Douglas and Thursday Island (Australia). The map demonstrates the spread of the four testing sites, indicating that the observed correlation patterns cannot be attributed solely to an unfortunate “bad” geographic location. The partial autocorrelation functions, ${\hat{\rho }}_{p}\left(h\right)$, for latitude, longitude and altitude are shown on the left for the four geographical locations as a function of the lag, *h*, measured in seconds.

The data on which we construct and test our methods were recorded by a single-frequency GlobalSat BU-353S4 unit operating on the L1 frequency, 1575.42 MHz [17]. We used this GPS receiver, fixed in position at one location in Dunedin, New Zealand, to record seven time-series sampled every second over time scales between approximately one and 25 days between the 6 August and the 24 November 2014. The same receiver recorded four additional time-series while fixed in position in three locations in Australia: North Stradbroke Island, Port Douglas and Thursday Island (see Figure 1). These additional time-series were also sampled every second and had time scales between approximately one and three days. The variety of locations and recording dates allowed us to check for possible geographical and temporal variation in the GPS signal, e.g., due to the varying position of GPS constellations. Additional data were taken at the same times and locations using tag-style GPS units, which also used the L1 frequency. This additional data had the same noise characteristics as the original data [8], showing that the noise properties, which we analyse below, are not dependent on the particulars of the GPS receiver, but rather attributed to environmental effects. The results in this paper are therefore expected to be applicable to any single-frequency GPS device operating on the L1 frequency.

### 2.1. Noise Correlations

**Figure 2:**
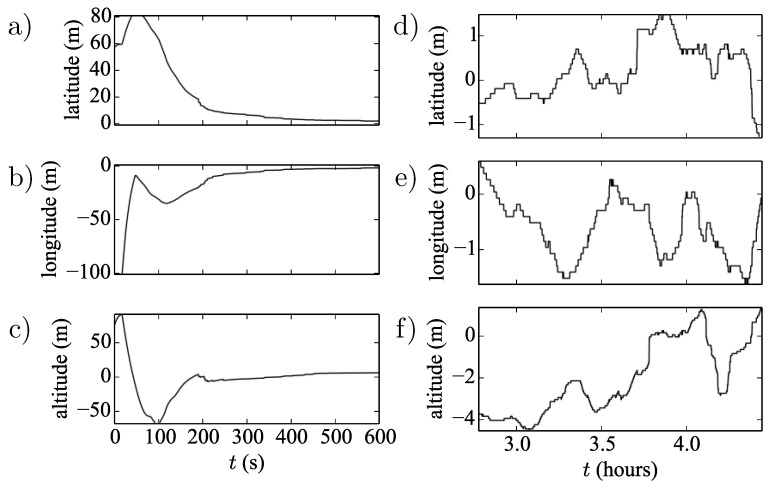
(**a**–**c**) First 600 s of GPS time-series residuals taken at Port Douglas, illustrating the rapid initial convergence towards the “true” value; (**d**–**f**) corresponding later GPS time-series residuals illustrating the time-correlated deviations from the “true” value (zero). The apparent position quantization due to rounding is also visible.

**Figure 3:**
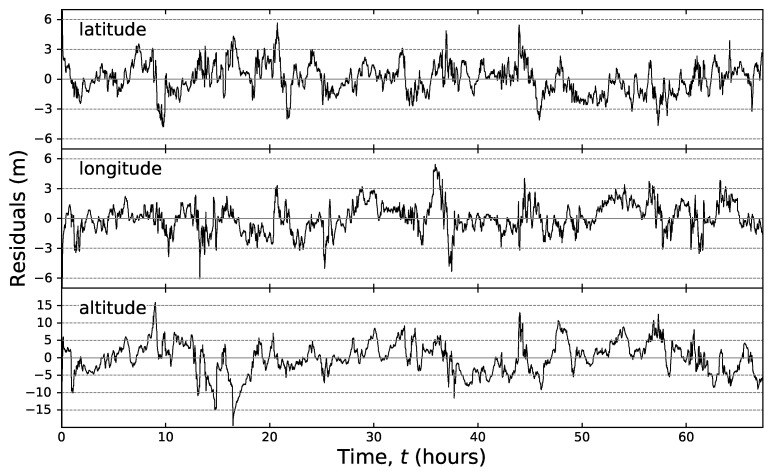
Residuals of latitude, longitude and altitude time-series at Port Douglas, Australia. The grey dashed lines guide the eye and highlight the mean-reverting nature of the time-series.

The datasets were analysed in detail in Reference [8], Here, we briefly present the findings relevant for this study. When positioned in a new location, the GPS device gives readings offset from the correct location over a distance scale of approximately 100 m. The readings tend to the correct location within a time scale of seconds to minutes. In this paper, all further analysis and inference is performed on datasets with the first 300 s truncated in order to ignore this initial transient behaviour. Subsequently, the readings take continual excursions with typical length scales of metres around what we assume to be the “true” location (see Figure 2 and Figure 3). These excursions have typical time scales of minutes to hours, causing the data to be autocorrelated [8]. Note that the GPS data are recorded in degrees to eight significant figures; these data are converted to meters, which, at the locations analysed in this paper, leads to an apparent quantisation of approximately 0.1 m. This quantised length scale is larger than the typical length scales for the processes, and thus, the data apparently jump between these quantised locations in tens to hundreds of seconds. None of the further treatment in this paper considers this apparent quantisation, which may be an avenue for further work.

### 2.2. Noise Models

We studied and evaluated the performance of various noise models for GPS data [8]; specifically, we focused on uncorrelated noise, OU, autoregressive processes ($x_{t}=-\sum_{k=1}^{p}h_{k}x_{t-k}+E_{t}$), moving-average processes ($x_{t}=\mu -\sum_{\ell =0}^{q}g_{\ell }E_{t-\ell }$) and their combination, an ARMA (p=1, q=1) model ($x_{t}=-h_{1}x_{t-1}+g_{0}E_{t}+g_{1}E_{t-1}$). Here, $x_{t}$ represents the signal at time-step *t*, while $E_{t}$ denotes the noise at the same time-step. The coefficients $h_{1}$, $g_{0}$ and $g_{1}$ are constants specific to the system under investigation. Using the standard Akaike Information Criterion (AIC) [18], we compared these model.

One of the conclusions of the comparison was that all noise models proved to be better than the the uncorrelated noise model. In this paper, we continue our investigations and compare techniques including or omitting correlation in noise models. An OU process is also included in these analyses, because it has the following beneficial properties: mean-reverting, continuous and having only a few parameters; hence, it lends itself to fast embedded computation and sensor fusion applications.

The OU process is defined by the stochastic differential equation:

(1) $$dx_{t}=\theta \;\left(\mu -x_{t}\right)\;dt+\sigma dW_{t},$$

where $W_{t}$ represents a Wiener process, while θ, μ and σ are parameters to be fitted. The associated Fokker–Planck equation has an analytical solution [11]:

(2) $$x_{t}∼N\left(\mu +\;\left[x_{0}-\mu \right]e^{-\theta t},\frac{\sigma^{2}}{2\theta }\left[1-e^{-2\theta t}\right]\right).$$

Here, $N\left(\mu_{n},\sigma_{n}^{2}\right)$ is the Gaussian distribution with mean $\mu_{n}$ and variance $\sigma_{n}^{2}$. This probability distribution for $x_{t}$ is initially a delta function located at $x=x_{0}$, with the limit, $N(\mu ,\sigma^{2}/\left(2\theta \right))$, as t→∞. The process is mean-reverting for θ>0; i.e., its limit is a stationary solution to the Fokker–Planck equation with mean μ, as t→∞. The time-discretised version of (1) for time-step Δt is given by:

(3) $$x_{t+1}=\mu +\;\left(x_{t}-\mu \right)\;\exp\;\left(-\theta \Delta t\right)\;+\;\epsilon_{t},$$

where $\epsilon_{t}$, the noise term, follows a Gaussian distribution with zero mean and variance $\frac{\sigma^{2}}{2\theta }\left[1-e^{-2\theta \Delta t}\right]$.

**Figure 4:**
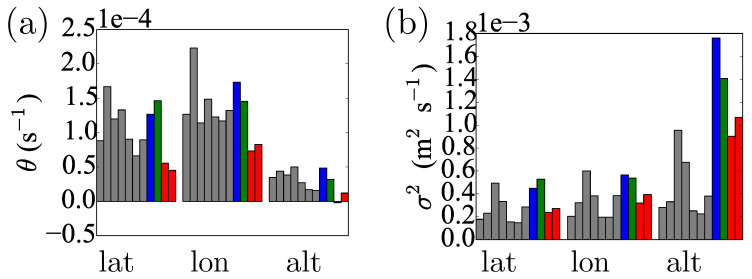
Fitted Ornstein–Uhlenbeck (OU) parameters to GPS time-series from Dunedin, New Zealand (grey), North Stradbroke Island, Australia (blue), Port Douglas, Australia (green), and Thursday Island, Australia (red). (**a**) Estimates of θ show no significant variation over geographic locations; however, the difference between the types of coordinates is apparent, θ being much smaller for altitude (alt) than for either latitude (lat) or longitude (lon). (**b**) Estimates of parameter $\sigma^{2}$ are depicted and show a similar pattern to θ in (**a**), although, here, the altitude is much larger than the other two coordinates. Qualitatively, this can be understood by the larger variability of the altitude residuals (see Figure 3).

During data processing, we assumed that the long-term average represents the “true” value for a random variable; hence, after subtracting this long-term average, the residual data must have zero mean, μ=0. Hence, from each residual, we obtained maximum likelihood estimates $\sigma^{2}$ and θ. For each coordinate, the logarithms θ and $\sigma^{2}$ were independently modelled, where the coordinate type (latitude, longitude and altitude) was assumed to have a fixed effect, while the geographic location and time have random effects. The geographic location seemed to have no significant effect on θ, but it weakly influenced $\sigma^{2}$; though, we believe, that this effect has no practical significance. The type of coordinate, on the other hand, seems to affect both θ and $\sigma^{2}$. Parameter θ was statistically significantly smaller for the altitude than for the other two coordinates; simultaneously, $\sigma^{2}$ was significantly larger for altitude, than for latitude or longitude. These results essentially confirm the conclusions of a visual inspection of Figure 4. Other models: higher order Autoregressive (AR), Moving-Average (MA) and, their combination, ARMA processes [8] are outside the scope of the current paper, but are promising candidates for the extension of this study (see Section 6).

## 3. Position and Uncertainty Estimation Methods

Before introducing Bayesian inference methods, we create a basic model that uses the raw position as the centre of a prediction interval, while the OU process determines the interval width. Specifically, our prediction for latitude, longitude and altitude are Gaussian distributions with variances $\sigma^{2}/\theta$, where σ and $\theta^{2}$ are the means of the fitted OU parameters for all three coordinates observed in Dunedin (see Section 2.2). We consider this model basic, because previous data do not inform later position estimates, hence cannot take into account the noise correlations. By using the parameters derived from the Dunedin time-series for the uncertainty quantification on the Australian time-series, we tested that the method works for datasets independent of those on which it was developed.

Receivers also often report DOP figures, horizontal and vertical, for the latitude, longitude and altitude coordinates. These numbers, being related to the diagonal elements of the covariance matrix, carry some limited information about time correlation. We incorporated this complementary data-stream into the statistical modelling. In Section 5, we present the effect of the HDOPand VDOPnumbers of the parameter estimates. If these estimates improve, DOP parameters are worthwhile to include in the statistical modelling of the noise.

### 3.1. Earlier Work

Since the late 1990s, few researchers outlined alternative ways to estimate distributional parameters of time-series of GPS signals and thereby analysed the noise inherent in these signals [19,20,21,22]. Johnson and Agnew even demonstrated, although for crustal velocities, that temporal correlation in observation can decisively influence quantities estimated from the measurement [23]. In general, the temporal correlation reduces the uncertainties of the geophysical parameters inferred from the signal; thus, one can be more certain of a potentially incorrect estimate if the temporal correlation is not considered. Most approaches relied on Maximum Likelihood Estimators (MLE) either in fitting ad-hoc models to the data or in estimating the covariance matrix of the signal components [19,24,25,26]. This MLE approach was considered to provide robust results [27] compared to linear regression. Similar to Johnson’s earlier study, Santamaría-Gómez et al. [28] and Masson et al. [29] concluded that incorrectly modelled noise in synthetic time-series introduces bias in the velocity data, and even worse, increasing the length of the time-series does not guarantee diminishing bias.

While some steps have been made to depart from the use of the white noise model, e.g., replacing it with time-correlated (i.e., coloured) noise [19,30], it is still often assumed that the noise can be modelled by independent, identically distributed Gaussian random variables. Bos et al. [21] developed a technique that substantially increases the efficiency of the MLE methods, which has since been improved [25]. Other, more advanced and computationally costly methods, e.g., Markov chain Monte Carlo methods [22,31] or wavelet analysis [32], have also been proposed to obtain non-biased probability distributions for the noise within the time-series.

### 3.2. Kalman Filter with Higher Order Noise

We formulate sophisticated methods that use Bayesian inference of the position and its uncertainties. Kalman filters [4] are a type of sequential Bayesian inference algorithms, often useful for real-time state estimation. An improved version, the Unscented Kalman Filter (UKF), allows state estimations on nonlinear systems by approximating the prior distribution by well-chosen “sigma” points [33,34]. The UKF, like other Kalman filters, assumes that a state, $x_{n}$, changes between two consecutive measurements, and this change can be described $f:x_{n}\mapsto x_{n+1}$, called the forward map. Furthermore, the measured quantities fully determine the state x, i.e., $g:x\mapsto \hat{y}$. Since one’s knowledge of the system of interest is incomplete, one often models it by a stochastic process, in which a random variation, known as process noise, is also added to the forward map. Likewise, g may also be contaminated by noise, representing random measurement errors. The Augmented-state UKF (AUKF) is a variant of the UKF, which describes the measurement and process noise distributions by “sigma” points [33,34,35,36,37]. The AUKF is described in Algorithm A1 located in Appendix A. Most Kalman filter schemes require the process and measurement noise to be additive; however, within the AUKF, the forward map and observation map are allowed in any form. The AUKF is therefore sufficiently flexible to handle quite general processes. Such processes include simultaneous estimation of OU parameter θ, which we can constrain to be positive by estimating its logarithm, and possibly moving-average processes [8,33,38,39,40], although moving-average processes would require a different choice of sigma points from those described in Algorithm A1. The AUKF will also be able to handle nonlinear forward maps due to some complicated sensor dynamics.

#### 3.2.1. Uncorrelated Gaussian Noise

For a GPS measurement with iid Gaussian noise (both in different coordinates and in time), the system state is represented by one position coordinate for each spatial direction considered (latitude, longitude and altitude). We, therefore, treat the coordinates separately: x=x; hence, the forward map, f, is the identity with vanishing process noise. The measurement noise is considered to be additive and uncorrelated: g(x)=x+ϵ, with $\epsilon ∼N(0,\Sigma_{v})$, and $\Sigma_{v}$ is associated with a stationary OU distribution $\Sigma_{v}=\sigma^{2}/\theta$. Here, $\sigma^{2}$ and θ are the same as for the non-learning method above.

At the start of the data processing, we initialise the prior distribution with state mean $\mu_{0}$ and covariance $K_{0}$. We chose $\mu_{0}$ to be 5 m from the “true” position, and the variance was taken to be $K_{xx,0}=20$ m^2^. This choice was motivated by the fact that for a Gaussian prior distribution, the true mean value lies near the edge of the central 90% probability region, since $\sqrt{K_{xx,0}}\approx 4.47$ m. An inference algorithm, we expect, would lead to a narrower posterior distribution around the true value.

The above procedure is used generally if a model diverges from the “true” value even if evolved by the exact dynamics. In such a case, some additive process noise is incorporated into the inference process. For the iid Gaussian model, the usage of such complementary process noise is equivalent to a Brownian motion around the “true” position. In order to find an optimal value for the process noise, we vary its magnitude and carry out inference with these Brownian models. Although these Brownian models contain correlated noise, their performance is inferior to a well-constructed OU model.

#### 3.2.2. Ornstein–Uhlenbeck Noise

Correlated noise can be included in the Kalman filter schemes by extending the state vector to include one or more parameters to represent the noise model. When an OU process generates the noise in the state variable, x is augmented by a new variable, $x_{t}$, determined by the OU process. The corresponding forward and measurement maps are $x_{t}\mapsto \;\left(x_{t}-\mu \right)\;\exp\;\left(-\theta \Delta t\right)\;+\;\epsilon$, where $\epsilon ∼N\left(0,\sigma^{2}\left(1-e^{-2\theta \Delta t}\right)\;/2\theta \right)$ and $g\left(x\right)=x_{t}$. The noise is thus shifted to the process noise from the observation noise (cf. Section 3.2.1); hence, the former can now be treated as inconsequential. Specifically, we choose a mean position coordinate μ and variance $K_{\mu \mu ,0}$ with the same values as *x* and $K_{xx,0}$ in the prior for the uncorrelated noise model. The state also includes the OU variate *x*; we chose a prior for *x* equal to the first data point of the GPS time-series and therefore started the filtering from the second data point onwards. Consequently, we chose a small value for the prior variance in the noise parameter $K_{xx,0}=0.1$ $m^{2}$. We also included the reversion rate parameter θ (or, more specifically, its logarithm) in the state, with the prior mean/variance of θ equal to the mean/variance of the fitted parameters for the Dunedin GPS datasets for latitude, longitude and altitude, respectively (see Section 2.2). Therefore, the inferences for the North Stradbroke Island, Port Douglas and Thursday Island time-series (which had no role in the parameter fitting) serve as a test of the general applicability of the algorithm in independent locations.

It is not possible to estimate the OU variance parameter, $\sigma^{2}$, using orthogonal sigma point selection for which the covariances of the state parameters with the process noise are set to zero (see Algorithm A1, Step 2). Hence, here, $\sigma^{2}$ was set for each coordinate as the mean of the fitted $\sigma^{2}$ for the Dunedin GPS datasets. The simultaneous estimation of $\sigma^{2}$ with the state parameters is left for future research.

## 4. Quantifying the Filtering Algorithm Performance

Similar to the method in Section 3.2.1, which issues time-series estimates of position in the form of Gaussian distributions, the UKF produces $\left\{x_{n}\right\}$ together with $\left\{\Sigma_{n}\right\}$. We interpret these as the mean and variance parameters of Gaussian posteriors. With this interpretation, the marginal distributions $\left\{q(x)\right\}$ of position are also Gaussian.

To compare and rank algorithms with different noise models according to their performance, we introduce the log-score [15,16,41] (ℓ=1 m):

(4) S(q|x)=−log(q(x)ℓ).

This quantity is often interpreted as a “surprise” of true value *x*. If *q* is large around the true value, the corresponding log-score, *S*, is low, i.e., the outcome is not surprising. An algorithm producing lower log-scores will be regarded as superior compared to those achieving higher *S* values.

## 5. Results

**Figure 5:**
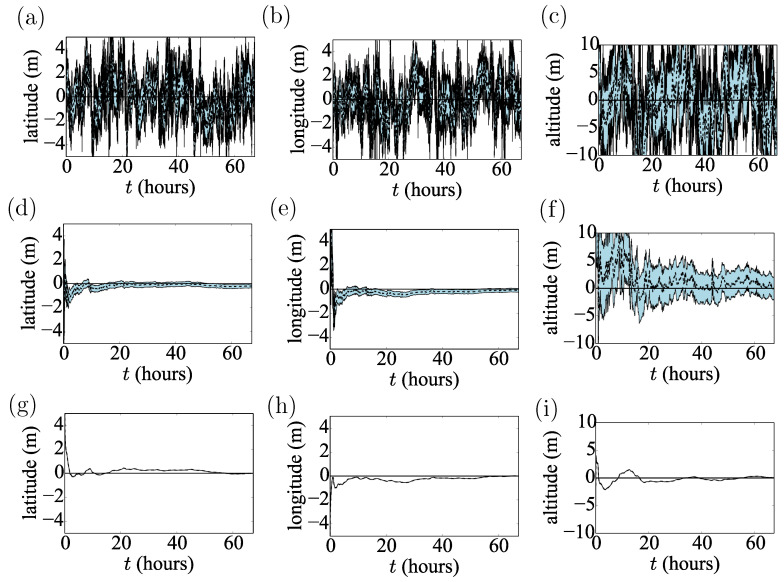
(**a**–**c**) The residuals are plotted for latitude (**a**), longitude (**b**) and (**c**) altitude as measured by a stationary receiver at Port Douglas. (**d**–**f**) The time-series of the inferred coordinates by an unscented Kalman filter algorithm equipped with OU noise model. The dataset plotted in (**a**–**c**) is used by this algorithm. Shaded regions represent the 90% credible intervals. (**g**–**i**) The same as the middle row, but using a Brownian model with process noise $\Sigma_{d}=1$ m^2^ within the UKF.

We analyse the predictions of the methods described in Section 3. Figure 5 shows the estimates for GPS data collected in Port Douglas. We note that this dataset is independent of the Dunedin datasets on which the model parameters were based. Port Douglas is a representative example, and the discussion below could be repeated for the other locations as well.

**Figure 6:**
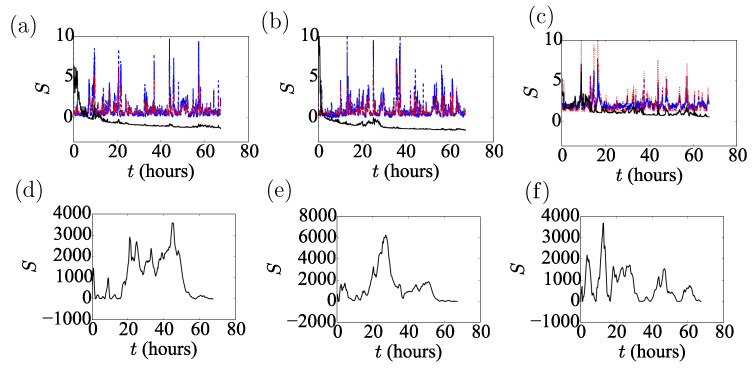
(**a**–**c**) Log scores, *S*, for latitude (**a**) longitude (**b**) and altitude (**c**) inferences from the Port Douglas data using the KF with the OU noise model. (**d**–**f**) The same as above, but for the KF with iid Gaussian noise model.

**Figure 7:**
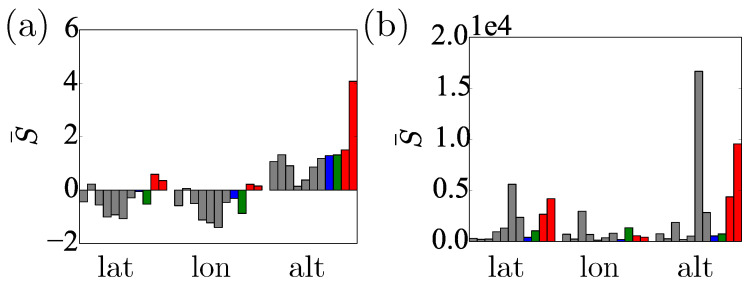
Performance of the predictions of the Unscented Kalman Filter with (**a**) the OU noise model and (**b**) iid Gaussian noise. Note that the $\bar{S}$ scale in (**b**) is $3\times 10^{3}$ bigger than in (**a**).

The uncertainty of the unprocessed location data is well captured by the Gaussian prediction obtained from the OU stationary distribution. Taking into account the reported DOP parameters led to small modifications only. Although occasional large spikes did appear in the uncertainties, these did not seem to correspond to large deviations in the position data. The time-series of the log scores of these predictions with and without DOP are very similar; cf. Figure 6d–f. Figure 7a,b shows the time-averaged log scores, $\bar{S}$, for both methods to be very similar, which holds for all the locations tested.

**Figure 8:**
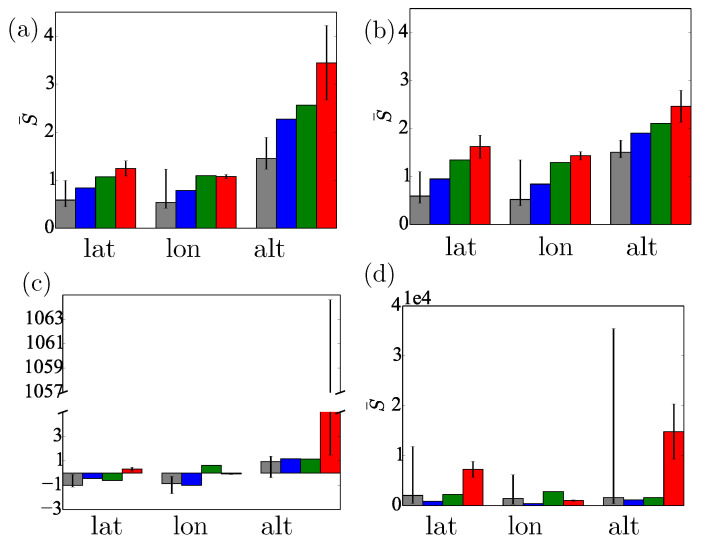
The time-averaged scores, $\bar{S}$, are plotted (**a**) for the raw data with the standard deviation given by the stationary limit and (**b**) for the same dataset as in (**a**) except that the uncertainty is multiplied by the appropriate Dilution-Of-Precision (DOP) variable. (**c**) $\bar{S}$ from the unscented Kalman filter with OU noise model and (**d**) the unscented Kalman filter with iid Gaussian noise model. The colours are consistent with Figure 4. The scales of the ordinates are different in each figure; most importantly, in (**d**), it is $3\times 10^{3}$ bigger than in the other figures.

The raw location data jump around the correct value, similarly to the marginals from the UKF inference obtained assuming a Brownian model. Due to the process noise, the Brownian model does not converge to the correct value. On the other hand, the position marginals from the UKF augmented with OU noise converge to the “true” value (see Figure 5d–f). The improvement in performance is supported by the log-scores. The posteriors produced from the OU noise score better by three orders of magnitude (see Figure 6). It is clear that the KF with the OU noise model is the superior method and the only method to produce negative scores, which continue to decrease with time. Figure 8 shows the scores for all the time-series from all locations averaged over the run-time. With respect to this average score, all KFs with OU noise outperform all other models using iid Gaussian noise models and all inferences using the non-learning method. The single exception is the altitude measurement for Thursday Island. This discrepancy is due to mischaracterised noise; $\sigma^{2}$ in this run was much larger than the one calculated from the Dunedin dataset. We thus have doubled $\sigma^{2}$ in the OU model for all the inferences. This manual intervention brought σ closer to the fitted value for the Thursday Island datasets, having negligible effect on the other inferences. We, therefore, suggest that extending the UKF to simultaneously estimate $\sigma^{2}$ during the inference process, in an embedded calculation, could allow the algorithm to adjust automatically to the variation of $\sigma^{2}$ with time and location.

As expected based on Figure 3 and Figure 4, the inferences of altitude are worse than those of the latitude and longitude. We believe that the otherwise well-characterised noise provides close to optimal quantification of uncertainty for the altitude coordinate, especially if one takes into account the intrinsic character of the altitude time-series due to the relative position of the satellite constellation.

**Figure 9:**
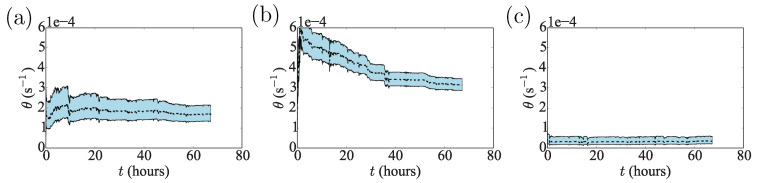
Estimates of the OU rate parameter θ for the (**a**) latitude, (**b**) longitude and (**c**) altitude time-series of a stationary receiver at Port Douglas.

We include for completeness the θ marginals in Figure 9. The θ estimates from the Bayesian filtering have the same orders of magnitude as the fitted parameters (see Figure 4), with the θ for the altitude coordinate smaller than the others, as expected. However, the θ for the longitude is larger than that for the latitude, which is not expected from the fitted version—it is perhaps an artefact of the imperfect model fit.

## 6. Discussion and Future Work

We developed algorithms for the sequential inference of the location of a GPS receiver, taking into account the correlated nature of the GPS noise. We found that the DOP parameters do not help in uncertainty quantification of the GPS positioning. This noise, which may be attributable to atmospheric and multipath effects, as well as the satellite configuration, is found to be correlated in time and can be modelled as an OU process. The AUKF algorithm using an OU noise model provides an accurate series of posterior distributions for GPS position with improved uncertainty quantification compared to inference assuming iid Gaussian noise provided a large enough value is assumed for the OU noise variance. The improved posterior distributions offer many practical advantages in applications in which knowledge of the correct uncertainty is important, e.g., automated navigation, where one must be very confident of avoiding obstacles. The algorithm is also easily extendible to incorporate data from additional sensors, e.g., accelerometers and gyroscopes to inform the inference (so-called sensor fusion). We expect that using a correlated model for the GPS noise in a sensor fusion application will extend its potential to improve accuracy, as a well-quantified position uncertainty will allow position corrections from other sensors to be accepted.

There are clear avenues for further improvement of the techniques in this paper. An algorithm similar to Algorithm A1 may be used to simultaneously estimate the OU variance $\sigma^{2}$ during the inference process, by including $\sigma^{2}$ as a state parameter. However, such an algorithm requires a different choice of sigma points from that in Algorithm A1. Additional sigma point should be added to the scheme with mixed components from $\sigma^{2}$ and the process noise, such that the mean and co-variance of the sigma points does not change. Furthermore, since we have earlier showed that more sophisticated noise models, e.g., the higher order AR processes and ARMA processes could be superior to the OU model, we expect that upgrading the noise models will lead to further improved position inference. The AUKF algorithm presented could be straightforwardly upgraded to use the higher order AR processes, taking care to parametrise the model such that it represents a stationary process. The forward map of an ARMA process also includes multiplicative noise, so to use such a forward map, similar care must be taken with the choice of sigma points.

We mention an extension of our current work to engineering applications. The effect of time-correlated noise on the applicability of classical Kalman filters has been previously analysed to a certain degree [42]. The noise models proposed here, however, can be seamlessly incorporated into a Kalman filter or Bayesian inference algorithm providing both an estimate for the location and the associated uncertainty quantified. Although such extensions require extra numerical calculation, the computational load is acceptable and provides seamless integration for sensor fusion, treating all sensors on equal footing. Incorporating the aforementioned noise models into such algorithms is in preparation.

Finally, although for the sake of transparency and simplicity, we modelled latitude, longitude and altitude separately as one-dimensional time-series, a more natural approach could be to combine them into one vector quantity. Building an inference algorithm similar to that in Section 3.2 would lead to a compact model that calculates and takes into account the covariances between these coordinates. This could further improve the algorithm performance.

## 7. Conclusions

We collected location data with a stationary GPS sensor at four locations: Dunedin (New Zealand) and North Stradbroke Island, Port Douglas and Thursday Island (Australia). The durations of these time-series range from a day to 25 days. We found appreciable autocorrelation within the noise present in the signal; thus novel, noise-models were constructed and compared for performance. In order to rank the performance of different noise models, we used the log-score. We found that an enhanced Kalman filter augmented with the Ornstein–Uhlenbeck noise model performed the best, and its pseudocode is provided. The computational cost of the corresponding algorithm is modest, and it generalises to sensor fusion application easily; thus, it is promising for embedded computing.

## Appendix A. Augmented-State UKF Algorithm

**Algorithm A1:** Augmented-state UKF [33,34,37].

## References

[1] Kuhlmann H. Kalman-filtering with coloured measurement noise for deformation analysis. Proceedings of the 11th FIG Symposium on Deformation Measurements.

[2] Li L., Kuhlmann H. (2012). Real-time deformation measurements using time-series of GPS coordinates processed by Kalman filter with shaping filter. Surv. Rev..

[3] Jo K., Chu K., Sunwoo M. (2012). Interacting Multiple Model Filter-Based Sensor Fusion of GPS with In-Vehicle Sensors for Real-Time Vehicle Positioning. IEEE Trans. Intell. Transp. Syst..

[4] Kalman R.E. (1960). A New Approach to Linear Filtering and Prediction Problems. J. Fluids Eng..

[5] Bryson A., Johansen D. (1965). Linear filtering for time-varying systems using measurements containing colored noise. IEEE Trans. Autom. Control.

[6] Bucy R. (1967). Optimal filtering for correlated noise. J. Math. Anal. Appl..

[7] Johnson D.J. (1970). Application of a colored noise Kalman filter to a radio-guided ascent mission. J. Spacecr. Rocket..

[8] Soundy A., Panckhurst B., Molteno T. Enhanced noise models for GPS positioning. Proceedings of the 6th International Conference on Automation, Robotics and Applications (ICARA).

[9] Petovello M.G., O’Keefe K., Lachapelle G., Cannon M.E. (2009). Consideration of time-correlated errors in a Kalman filter applicable to GNSS. J. Geod..

[10] Jiang P., Zhou J., Zhu Y. Globally optimal Kalman filtering with finite-time correlated noises. Proceedings of the 49th IEEE Conference on Decision and Control (CDC).

[11] Uhlenbeck G.E., Ornstein L.S. (1930). On the Theory of the Brownian Motion. Phys. Rev..

[12] Jolliffe I.T., Stephenson D.B. (2003). Forecast Verification: A Practitioner’s Guide in Atmospheric Science.

[13] Bröcker J., Smith L.A. (2007). Scoring Probabilistic Forecasts: The Importance of Being Proper. Weather Forecast..

[14] Boero G., Smith J., Wallis K.F. (2011). Scoring rules and survey density forecasts. Int. J. Forecast..

[15] Gneiting T., Ranjan R. (2011). Comparing Density Forecasts Using Threshold- and Quantile-Weighted Scoring Rules. J. Bus. Econ. Stat..

[16] Martin A.D., Molteno T.C.A., Parry M. Measuring the performance of sensors that report uncertainty. Proceedings of the 21st Electronics New Zealand Conference.

[17] Martin A.D., Soundy A.W.R., Panckhurst B.J., Brown C.P., Schumayer D., Molteno T.C.A., Parry M. Real-time uncertainty quantification using correlated noise models for GNSS positioning. Proceedings of the 2017 IEEE SENSORS.

[18] Akaike H. (1974). A new look at the statistical model identification. IEEE Trans. Autom. Control.

[19] Mao A., Harrison C.G.A., Dixon T.H. (1999). Noise in GPS coordinate time-series. J. Geophys. Res. Solid Earth.

[20] Kim D.M., Suk J. (2012). GPS output signal processing considering both correlated/white measurement noise for optimal navigation filtering. Int. J. Aeronaut. Space Sci..

[21] Bos M.S., Fernandes R.M.S., Williams S.D.P., Bastos L. (2013). Fast error analysis of continuous GNSS observations with missing data. J. Geod..

[22] Bos M.S., Montillet J.P., Williams S.D.P., Fernandes R.M.S., Montillet J.P., Bos M.S. (2020). Introduction to Geodetic Time Series Analysis. Geodetic Time Series Analysis in Earth Sciences.

[23] Johnson H.O., Agnew D.C. (1995). Monument motion and measurements of crustal velocities. Geophys. Res. Lett..

[24] Langbein J. (2004). Noise in two-color electronic distance meter measurements revisited. J. Geophys. Res. Solid Earth.

[25] Langbein J. (2017). Improved efficiency of maximum likelihood analysis of time-series with temporally correlated errors. J. Geod..

[26] Williams S.D.P., Bock Y., Fang P., Jamason P., Nikolaidis R.M., Prawirodirdjo L., Miller M., Johnson D.J. (2004). Error analysis of continuous GPS position time-series. J. Geophys. Res. Solid Earth.

[27] Hackl M., Malservisi R., Hugentobler U., Wonnacott R. (2011). Estimation of velocity uncertainties from GPS time-series: Examples from the analysis of the South African TrigNet network. J. Geophys. Res. Solid Earth.

[28] Santamaría-ómez A., Bouin M.N., Collilieux X., Wöppelmann G. (2011). Correlated errors in GPS position time-series: Implications for velocity estimates. J. Geophys. Res. Solid Earth.

[29] Masson C., Mazzotti S., Vernant P. (2019). Precision of continuous GPS velocities from statistical analysis of synthetic time-series. Solid Earth.

[30] Caron F., Duflos E., Pomorski D., Vanheeghe P. (2006). GPS/IMU data fusion using multisensor Kalman filtering: Introduction of contextual aspects. Inf. Fusion.

[31] Olivares-Pulido G., Teferle F.N., Hunegnaw A., Montillet J.P., Bos M.S. (2020). Markov Chain Monte Carlo and the Application to Geodetic Time Series Analysis. Geodetic Time Series Analysis in Earth Sciences.

[32] Kaczmarek A., Kontny B. (2018). Identification of the Noise Model in the Time Series of GNSS Stations Coordinates Using Wavelet Analysis. Remote Sens..

[33] Wan E.A., van der Merwe R. The unscented Kalman filter for nonlinear estimation. Proceedings of the IEEE 2000 Adaptive Systems for Signal Processing, Communications, and Control Symposium.

[34] Julier S., Uhlmann J. (2004). Unscented filtering and nonlinear estimation. Proc. IEEE.

[35] Chan Y.T., Hu A.G.C., Plant J.B. (1979). A Kalman Filter Based Tracking Scheme with Input Estimation. IEEE Trans. Aerosp. Electron. Syst..

[36] Chang S.Y., Mills G., Latif S. (2012). Application of Kalman Filter with Time-Correlated Measurement Errors in Subsurface Contaminant Transport Modeling. J. Environ. Eng..

[37] Martin A., Molteno T. Automated weighing by sequential inference in dynamic environments. Proceedings of the 2015 6th International Conference on Automation, Robotics and Applications (ICARA).

[38] van der Merwe R., Wan E.A. The Square-Root Unscented Kalman Filter for State and Parameter-Estimation. Proceedings of the International Conference on Acoustics, Speech, and Signal Processing.

[39] Wang K., Li Y., Rizos C. (2012). Practical Approaches to Kalman Filtering with Time-Correlated Measurement Errors. IEEE Trans. Aerosp. Electron. Syst..

[40] Wang X., Ni W. (2016). An improved particle filter and its application to an INS/GPS integrated navigation system in a serious noisy scenario. Meas. Sci. Technol..

[41] Constantinou A., Fenton N. (2012). Solving the Problem of Inadequate Scoring Rules for Assessing Probabilistic Football Forecast Models. J. Quant. Anal. Sport.

[42] Wendel J., Trommer G.F. An Efficient Method for Considering Time Correlated Noise in GPS/INS Integration. Proceedings of the 2004 National Technical Meeting of the Institute of Navigation.

